# Quantitative and Qualitative Analysis of Medical Students’ Research Output in Five Developing Countries

**DOI:** 10.7759/cureus.8026

**Published:** 2020-05-08

**Authors:** Mohammad Abrar Shareef, Adam S Obad, Haneen T Salah, Abdulaziz M Eshaq, Judie Hoilat, Alaa Alsaffar, Abdulrahman M Bakather, Wedad Alnajjar, Ahmed M Fothan, Meryam Almedani, Abdulkarim Sulaihim, Khaled M Al-kattan, Abdulhadi A Alamodi

**Affiliations:** 1 Internal Medicine, Northern Light Sebasticook Valley Hospital, Pittsfield, USA; 2 Medicine, Alfaisal University, Riyadh, SAU; 3 Pathology and Laboratory Medicine, Alfaisal University, Riyadh, SAU; 4 Surgery, Alfaisal University, Riyadh, SAU; 5 Internal Medicine, Alfaisal University, Riyadh, SAU; 6 Internal Medicine, Jackson State University, Jackson, USA; 7 Oral and Maxillofacial Surgery, King Saud Medical City, Riyadh, SAU; 8 Epidemiology and Public Health, School of Public Health, Jackson State University, Jackson, USA

**Keywords:** impact factor, publication, research output, quantity, quality, dissemination

## Abstract

Objectives

The purpose of this analysis was to investigate the quantity and quality of medical students’ research output in Gulf Cooperation Council countries to aid in developing strategies to improve research output.

Methods

Abstracts presented by medical students in Gulf Cooperation Council countries were subject to analysis. Abstracts that propagated into full-length articles underwent further demographic analysis, in which data regarding the type of study, the field of study, country of origin, mode of presentation, and journal’s impact factor were collected. A total of 798 abstracts were surveyed, with 19% (n=155) of the abstracts submitted by Gulf Cooperation Council countries progressing into full-length publications. The average impact factor for Gulf Cooperation Council country publications was found to be 1.85 ± 0.26 (standard error). Countries that recorded the highest conversion rates were, in descending order, Kingdom of Saudi Arabia, United Arab Emirates, Oman, Bahrain, and Kuwait. Moreover, basic biomedical and clinical research topics were more likely to be published in comparison with community-oriented and medical education-related topics.

Conclusions

Effective efforts to encourage more medical student research output in the Gulf Cooperation Council countries (with a focus on qualitative analysis) should be promoted in order to achieve publication rates comparable with those reported by developed countries.

## Introduction

Training medical students to develop research competencies has been a focus of many medical schools worldwide [1]. Efforts have concentrated on tackling the noted decline in rates of physician-scientists. Physician-scientists play significant roles in accelerating the progress of bench to bedside translational research and, hence, advancing the future of health care [2]. A plausible solution recommended in the literature to overcome this obstacle is to engage medical students in research endeavors during their medical training. As an added benefit, allowing students to participate in research projects while in medical school would enable them to acquire a broad spectrum of invaluable research skills such as high levels of cognition (critical thinking, decision-making, and problem-solving) and effective communication [3-4]. Although medical students’ engagement in research is rewarding, some consider that research experiences are not complete unless they are culminated by the dissemination of knowledge. Knowledge dissemination can be expressed in two forms: (1) peer-reviewed research articles and (2) professional presentations in scientific conferences [5]. Publishing in peer-reviewed journals plays a significant role with regard to the employment of future physicians and has been suggested to be among the factors that predict whether a student will persist with an academic medicine career [6].

Several studies conducted in both developed and developing countries have addressed medical students’ perceptions, practices, and attitudes toward medical research. However, few studies incorporate objective measures, such as publishing rates, to estimate the outcome of medical students’ engagement in research experiences. Most studies show that 30% of students’ research in developed countries tends to be propagated into the literature [7]. To the best of our knowledge, there has been no large-based analysis of medical students’ research output (manifested by peer-reviewed publications as an objective measure) in developing countries.

Even though medical students’ research activities are not given equal importance in developing countries as compared with developed countries such as the United States, there has recently been an increasing interest in promoting medical students’ research activities in developing countries such as the Gulf Cooperation Council (GCC) countries (Kingdom of Saudi Arabia [KSA], Kuwait, United Arab Emirates [UAE], Qatar, and Bahrain). Specifically, efforts have been directed toward promoting further integration of research activities into medical curricula, organizing medical student conferences on national levels, devoting sections for medical students in indexed journals, and establishing independent bodies in institutions to serve student research activities [8-10]. The objective of our paper is to analyze medical students’ research output in the GCC countries by evaluating (1) conversion rates of research abstracts propagating into full-length articles in peer-reviewed journals and (2) factors that predict publication among medical students in these developing countries.

## Materials and methods

The GCC medical students’ conference is an important platform for the dissemination of medical student research projects, specifically in gulf countries. The conference is typically held every two years in one of the GCC countries, where hundreds of medical students present their research projects orally or as poster presentations in all clinical, medical education, community, and basic science-related topics. Also, the conference provides additional activities that help foster student skills and knowledge in various areas related to medical education. When initiating the current research paper, data from the first eight conferences were divided among the authors of the study to examine the abstracts. Conference no. 3 was not included in the analysis due to the unavailability of data on the website. Upon review of several reports in the literature in which similar types of studies were conducted, we have developed our methods into the following steps.

Abstract retrieval

A total of 798 abstracts were retrieved from the official website of the GCC medical students’ conference [11]. Each abstract was subject to inclusion and exclusion criteria. Abstracts not affiliated with a GCC country were excluded from the study in order to remain consistent with the objectives of the study.

Data entry development

Abstracts that met the inclusion criteria were subject to further analysis. A database was developed using Microsoft Excel 2013. Initially, each respective conference’s abstracts were analyzed independently. Demographic data including the field of study (clinical, basic biomedical, community-oriented, or medical education study), type of study (original article, review article, or case report and case series), country of origin, and mode of presentation were obtained.

Full paper extraction

All abstracts were investigated to determine the studies that had propagated into full published articles. In this step, we used two well-known search engines (PubMed and Google Scholar). Titles and keywords were employed in order to retrieve full papers. However, as titles are subject to change, we used the names of at least two authors to ensure the accuracy of the retrieval process. The first and corresponding author’s names were used in the PubMed search engine for every abstract. For those papers that progressed into full published manuscripts, we obtained the following information for qualitative assessment: (1) the journal’s name and (2) the journal’s impact factor.

Determining publication predictors

All abstracts that propagated into full published articles were copied and pasted into a Microsoft Word 2013 document to be compared with the ones presented in the conference. Different variables such as type of study, the field of study, mode of presentation, and country of origin were used to assess the predictability of abstracts propagating into full published articles in the literature.

Statistical analysis

The statistical analysis of this project was divided into three parts. The demographic characteristics (particulars) of the abstracts were determined using Microsoft Excel 2013, as previously indicated. Similarly, Microsoft Excel 2013 was used to calculate the overall conversion rate as well as conversion rates in relation to different variables. SPSS Statistics for Windows, Version 16.0 (SPSS Inc., Chicago, IL, USA) was used to examine the relationship between the rate of publication and studied variables. A multivariate binary logistic regression test was performed to determine the predictability of article dissemination by one of the following variables: field, type, country, or mode of the study. Additionally, the Cox proportional hazard model was conducted to identify the factors that play a role in delaying the publication of an abstract. Furthermore, the relationship between the impact factor of targeted journals and the time required for abstracts to fully progress into full-length articles was examined using bivariate Pearson’s correlation test.

## Results

Demographics of retrieved studies

**Figure 1:**
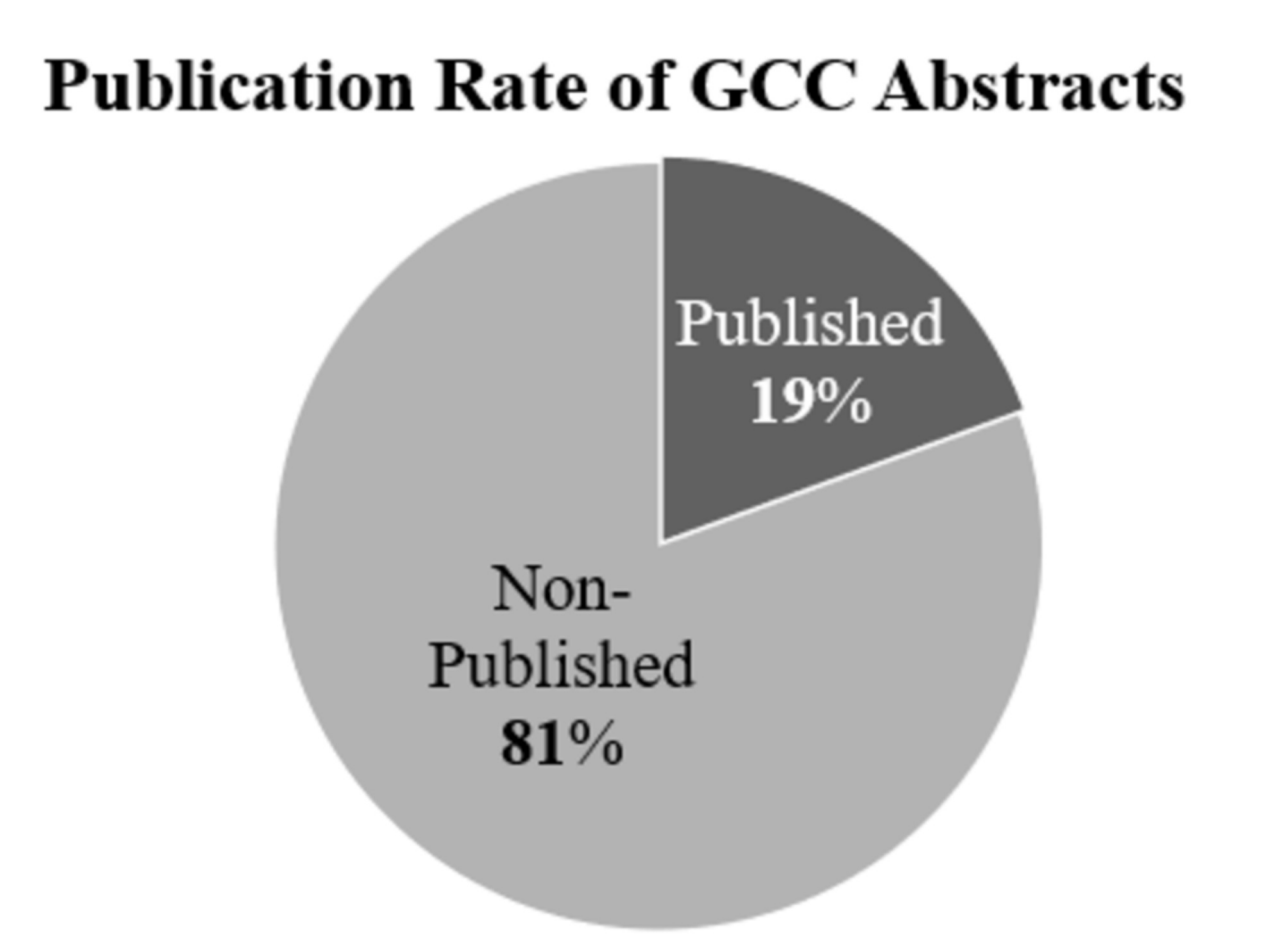
Percentages of published and non-published abstracts in GCC. GCC, Gulf Cooperation Council Countries

In total, 798 abstracts were retrieved from the official website of the GCC conference, from which only 155 abstracts propagated into full-length articles and were published in peer-reviewed journals (Figure 1), which is a conversion rate of 19%. Regarding the field of study, 196 abstracts were basic biomedical studies and 289 were clinical studies, with publication rates of 27% and 22%, respectively. Most of the studies came from the KSA (n=296) and UAE (n=217), with publication rates of 23% and 21%, respectively. Most of the studies were original (n=757), with only 21 review articles included at GCC conferences. Additionally, 360 articles were presented orally and 438 studies were presented as posters, with related publication rates of 20% and 10%, respectively. Table 1 displays the demographic characteristics of the retrieved abstracts.

**Table 1:** Demographic data of abstracts presented in the GCC conference. N represents the number of abstracts. KSA, Kingdom of Saudi Arabia; UAE, United Arab Emirates

	Type	Presented abstracts (N=798)	Published abstracts (N=155)
Field of study	Basic biomedical research	196	53 (27%)
	Clinical research	289	65 (22%)
	Community-oriented research	251	32 (13%)
	Medical education research	50	4 (8%)
	Other research studies	12	1 (8%)
Country	KSA	296	67 (23%)
	UAE	217	46 (21%)
	Oman	141	26 (18%)
	Bahrain	69	11 (16%)
	Kuwait	74	5 (7%)
Type of study	Original	757	150 (20%)
	Review	21	2 (10%)
	Case report and series	18	3 (17%)
Mode of presentation	Oral	360	82 (23%)
	Poster	438	73 (17%)

Demographics of published studies

Of all the published articles, 39% (n=57) were basic biomedical studies and 42% (n=62) were clinical studies. However, community-based and educational studies constituted 18% (n=26) and 2% (n=3), respectively. Moreover, while 54% (n=80) of the abstracts were presented orally, 46% (n=68) were poster presentations. The average publication time was 21.41 ± 1.85 (average number of month ± standard error [SE]). A total of 73 articles were published prior to their presentation at the conference. Upon merging all studies, the average publication time was estimated to be 15.97 ± 1.58 (average number of the month ± SE). Table 2 displays the demographic data of published research articles.

**Table 2:** Demographic data of medical students' published research articles. N represents the number of abstracts.

Variable	N (%)
Basic biomedical research articles	57 (39%)
Clinical research articles	62 (42%)
Community-based articles	26 (18%)
Medical educational articles	57 (39%)
Oral presentation	80 (54%)
Poster presentation	68 (46%)
Duration (months)	15.97 ± 1.58

**Figure 2:**
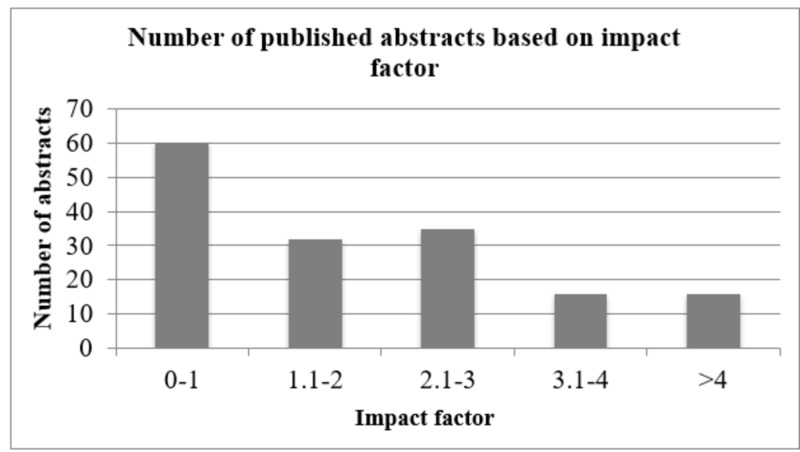
Number of published articles and the corresponding impact factor of journals.

The mean impact factor of the targeted journals was 1.85 ± 0.26 (SE). However, 40.5% (n=60) were published in journals with impact factors < 1 (Figure 2). Another set of abstracts (20.27% [n=30]) were published in journals with impact factors > 3. Furthermore, different types of research showed different impact factor values. While the mean impact factor for basic biomedical is 2.6 ± 0.21, it was 1.89 ± 0.21 for clinical studies. Community-oriented studies, on the other hand, had a mean impact factor of 0.99 + 0.19. Finally, educational studies had a mean impact factor of 1.89 ± 0.58. Figure 2 shows the impact factor corresponding to the number of published articles.

Multivariate binary logistic regression test

The results of the multivariate binary logistic regression test showed that significant predictors of publication into journals include both the field of study and the country of origin. Basic biomedical studies were more likely to be published when compared with both community-oriented and educational studies (p<0.001 and p=0.005, respectively). Additionally, abstracts originating from Bahrain and Kuwait had a lower chance for publication compared with abstracts originating from KSA (p=0.03 and p=0.01, respectively). Neither the type of the study nor the mode of presentation was a significant predictor of publication (p=0.61 and p=0.09, respectively). Table 3 displays factors associated with the likelihood of publication.

**Table 3:** Factors associated with medical students' publishing rates in the Gulf Cooperation Council countries. OR, odds ratio; CI, confidence interval; UAE, United Arab Emirates

Variable	Internal value (0)	Internal value (1)	p-Value (0.001)	Coefficient (B)	OR	95% CI
Field of study	Community	Basic biomedical	<0.001	-0.96	0.39	0.25-0.66
	Educational studies	Basic biomedical	0.005	-1.58	0.21	0.07-0.62
	Clinical research	Basic biomedical	0.20			
Country	Kuwait	Kingdom of Saudi Arabia	0.01	-1.3	0.28	0.11-0.74
	Bahrain	Kingdom of Saudi Arabia	0.03	-0.84	0.43	0.21-0.91
	UAE	Kingdom of Saudi Arabia	0.10			
	Oman	Kingdom of Saudi Arabia	0.09			
Mode	Poster	Oral	0.09			

Cox proportional hazard modeling

All factors, including the field of study, country of origin, type of research project, and the method of presentation, did not affect the publication time with p-values of 0.67, 0.09, 0.41, and 0.31, respectively. The significant finding from this analysis was that the abstracts originating from Oman took more time from the first presentation to publication as compared with the abstracts produced in KSA (p=0.005). However, there is no significant difference between abstracts from KSA and other gulf countries with regard to the period of dissemination.

## Discussion

This study was undertaken to assess, in quantitative and qualitative measures, the end outcome of medical students’ research experiences represented by their abstract presentations in the GCC conferences from 2004 to 2012 in five developing countries. Medical students’ research experiences are more rewarding and meaningful upon the culmination of research experiences through knowledge dissemination in the form of published manuscripts. One of the primary findings in this study is that 19% (n=148) of the presented abstracts (for all the GCC medical students together) were found to have progressed into full-length published articles in the literature (Figure 1). Several reports investigated the rates at which medical students tend to publish their research projects in developed countries. A study conducted in the United Kingdom found that 28% of the students’ research projects were published in the literature [12]. In Germany, approximately 66% of medical students’ research projects were found to be published [13]. However, reports from the United States have been found to have significantly higher conversion rates than those in the United Kingdom, Germany, and the Gulf region, with values of 41% and 90% in the Mayo Clinic and Stanford University, respectively [14-15]. However, the present publishing rate among medical students in the five GCC countries was not found to be dissimilar from reported numbers in other developing countries such as India, in which 20% of the medical students’ research experiences were culminated by the publication of manuscripts [13]. A recent meta-analysis revealed that, on average, 30% of medical students’ research projects tend to propagate into full-length articles [7]. This discrepancy between countries in the developing and developed world is not unexpected because medical students in developing countries are subject to more challenges and barriers in research activities. These challenges may include (1) a lack of proper infrastructure and research environment, (2) a lack of funding, and (3) a lack of accessibility to mentors [4,16-17].

Upon examination of individual countries in the Gulf region, it was revealed that KSA and UAE have the highest conversion rates of publications, with values of 23% and 21%, respectively. Following these countries are Oman and Bahrain, with conversion rates of 18% and 16%, respectively. Kuwait exhibits the lowest conversion rate, with values close to 7% (Table 1). The results exhibiting KSA and UAE’s higher conversion rates for publications indicate that the culture surrounding the publication of manuscripts is more supportive in these countries. KSA and UAE’s efforts toward engaging undergraduate medical students in rewarding and meaningful research experiences are more significant in comparison to other GCC countries. Countries such as Kuwait, Oman, and Bahrain should re-evaluate their undergraduate research programs and opportunities offered to their students to make them more rewarding and meaningful. It is important to note, however, that the potential reasons for the discrepancies may result from the sample used for analysis in our study and its protocol. Another potential indirect cause could be related to the amount of integration of research into teaching and the medical curriculum. Existing literature suggests that there is a growing interest in medical schools around the Gulf region to reform their medical curricula to that which supports learning and teaching in a research-based fashion such as the problem-based learning (PBL) strategy [13]. Indeed, undergraduate medical research and PBL have been closely linked, so much so that even students studying under a PBL-based curriculum perceive research better than those studying under a non-PBL-based curriculum [14]. Although publishing should not be the central driving force for students to participate in research programs, offering structured research opportunities that culminate in knowledge dissemination would recruit and retain medical students in undergraduate research programs and aid in the effort to reduce the crisis in declining physician-scientist rates [2,16,10].

Besides the quantitative aspect, some institutes value the qualitative aspect greatly. In our study, we used the impact factor of the published articles as a measure of the quality of research opportunities in which medical students were participating. It has been reported that most of the students’ published articles are not cited, which brings into question the validity of their results and calls for concern regarding their reliability [13]. Hence, the impact factors of targeted journals have been examined in similar types of studies. In our study, the mean impact factor was 1.85 ± 0.26 (SE), which was expected for such a multidisciplinary conference. However, 60 (42.5%) of the published abstracts were published in journals with impact factors lower than 1 (Figure 2). As reported previously, the average impact factor of journals that publish the articles of medical students in our institute was 2.4 [10]. We also recently reported a large analysis based on PubMed, indexed research articles by GCC country, and the average impact factor for all the GCC countries (which is 1.6) [18]. The current figure in this study is higher, demonstrating that medical students’ research articles do not differ in quality from those reported by faculty members in the GCC countries. There should be a call to improve the quality of research conducted in these developing countries. This could be achieved by dedicating conferences or workshops to address the current status of research in the GCC countries, creating mechanisms to enhance the quantity as well as the quality of GCC country research by training junior investigators and collaborating with international experts.

Another important aspect that our study attempts to address is the factors affecting knowledge dissemination of medical students’ abstracts into full published articles. Our study demonstrates higher publication rates in basic biomedical and clinical research compared with community-oriented and medical education research (p=0.001 and p=0.005, respectively). These findings were expected due to the nature of work in such different fields. For example, in the setting of laboratory- or hospital-based research projects, medical students often join a team of experts and work under the mentorship of a research scientist or physician-researcher, respectively. This is driven by the fact that such projects are funded by research associations and institutes, where publishing is among the criteria when funding is given. However, in the setting of community-oriented and medical education-related projects, medical students often conduct projects independently. They perform almost all research steps starting from generating a research idea to designing necessary tools, collecting data, and analyzing results. Even with individual efforts by some of the medical students when conducting community-oriented and medical education-related projects, many do not consider submitting their work to peer-reviewed journals. Students are often satisfied as long as they meet the course’s demands and achieve high grades. This finding, we believe, should alert institutes to call for changes that include dedicating human resources or “mentors” to help medical students publish their independent research projects in peer-reviewed journals. It can be argued that dedicating such resources requires extensive efforts. Incorporating the strategy of promoting senior medical students to serve as mentors to their junior colleagues would help increase the chances of establishing more effective research opportunities and decrease the load on the faculty and staff. Indeed, we previously reported that senior medical students could serve as effective mentors to junior medical students when conducting research courses [1,9-10].

Medical education has witnessed two reforms over the past century. In 1910, the Flexner Report called for effective integration of basic sciences into medical education, and in 1915, the Welch-Rose Report introduced PBL strategies into medical education curricula [19]. However, because of the evolution of medical education, there are still areas that exhibit gaps and need enhancement within many medical education curricula. Over the past three decades, institutions have been faced with obstacles in engaging medical students in research programs and activities. Unfortunately, this aspect of medical education remains substantially unstructured, especially in developing countries, which are highlighted in this study [4]. We have created a model of undergraduate research committees that we believe can assist in improving the current status of undergraduate medical research in the GCC countries if implemented effectively. Through developing an innovative, student-run, well-structured, and integrative independent research body, we were able to boost students’ outcomes and productivity in research. For example, before graduation of the first batch of medical students, 50% were able to publish research in peer-reviewed journals with an average impact factor of 2.4, which, when weighted, indicated that the reported figures in this study have a better outcome [10]. Other approaches to boost medical students’ research output include promoting student journals to be indexed in PubMed, engaging medical students in the review process, dedicating spaces for medical students in peer-reviewed journals, offering structured research experiences likely to result in research publication, promoting student confidence and independence in developing their research ideas, and helping students translate their research ideas into publishable material [7,10].

There are several limitations to this study. First, the analysis was conducted in five developing countries, and therefore we cannot generalize our data to the rest of the developing world. Second, the element of bias cannot be excluded; we selected a specific sample of abstracts that were presented in the students' conference. Also, we did not look at the time of presentation to the time of publication. Future studies require a wider base of analysis to assess the publications' practice among medical students in developing countries.

## Conclusions

The overall percentage of the abstract that propagated into full-text articles in the GCC countries was 19%. The majority of published manuscripts were in journals with an impact factor below 1. Abstracts from KSA and in the field of biomedical sciences seem to propagate more into full-length articles compared with other countries and types of research, respectively. Strategies should be tailored toward promoting engaging medical students in research experiences that ultimately lead to knowledge dissemination in the medical literature.
